# Kikuchi–Fujimoto disease manifesting bilateral lymphadenopathy

**DOI:** 10.1002/ccr3.6494

**Published:** 2022-10-20

**Authors:** Akihiro Kawatsuki, Kosuke Oka, Hideharu Hagiya, Fumio Otsuka

**Affiliations:** ^1^ Okayama University Medical School Okayama Japan; ^2^ Department of General Medicine Okayama University Graduate School of Medicine, Dentistry and Pharmaceutical Sciences Okayama Japan

**Keywords:** bilateral lymphadenopathy, Kikuchi‐Fujimoto disease

## Abstract

Physicians should keep in mind that Kikuchi‐Fujimoto disease can show bilateral lymphadenopathy like the present case.

## Graphical abstract

Kikuchi–Fujimoto disease (KFD) usually presents with unilateral lymphadenopathy. However, it is important to differentiate KFD from other diseases presenting with bilateral lymphadenopathy such as malignant lymphoma and infectious mononucleosis.

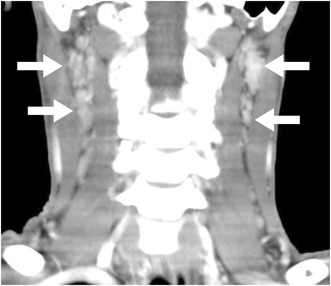

An 18‐year‐old man was transferred to our hospital for prolonged fever over 38°C with swelling of bilateral cervical lymph nodes (Figure 1, arrows). White blood cell count was reduced at 1600/ml with atypical lymphocyte percentage of 7%. Serum levels of lactate dehydrogenase (LDH) (1271 U/L), soluble IL‐2 receptor (1293 U/ml), ferritin (3065 ng/ml), aspartate aminotransferase (AST) (292 U/L), and alanine aminotransferase (ALT) (284 U/L) were elevated. Lymph node biopsy was performed, and the pathology showed patchy areas of necrosis, proliferation of pale histocytes, and increased apoptotic cells, supporting a diagnosis of Kikuchi–Fujimoto disease (KFD) (Figure 2). No proliferation of lymphocytes with dysplasia, which supports a diagnosis of malignant lymphoma, was observed. The persistent fever subsided spontaneously without any treatment.

**FIGURE 1:**
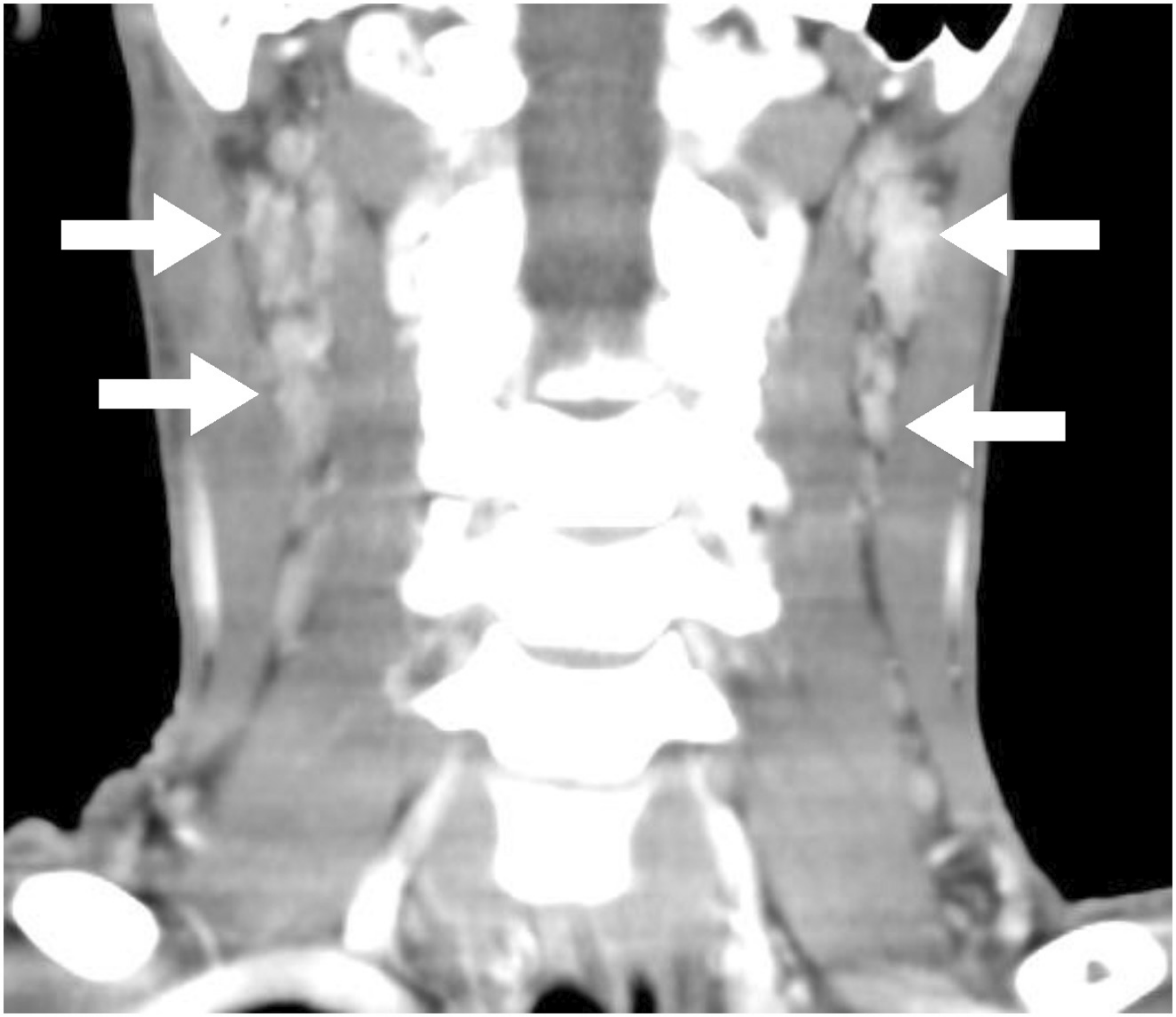
CT image of the bilateral neck lymphadenopathy (arrows)

**FIGURE 2:**
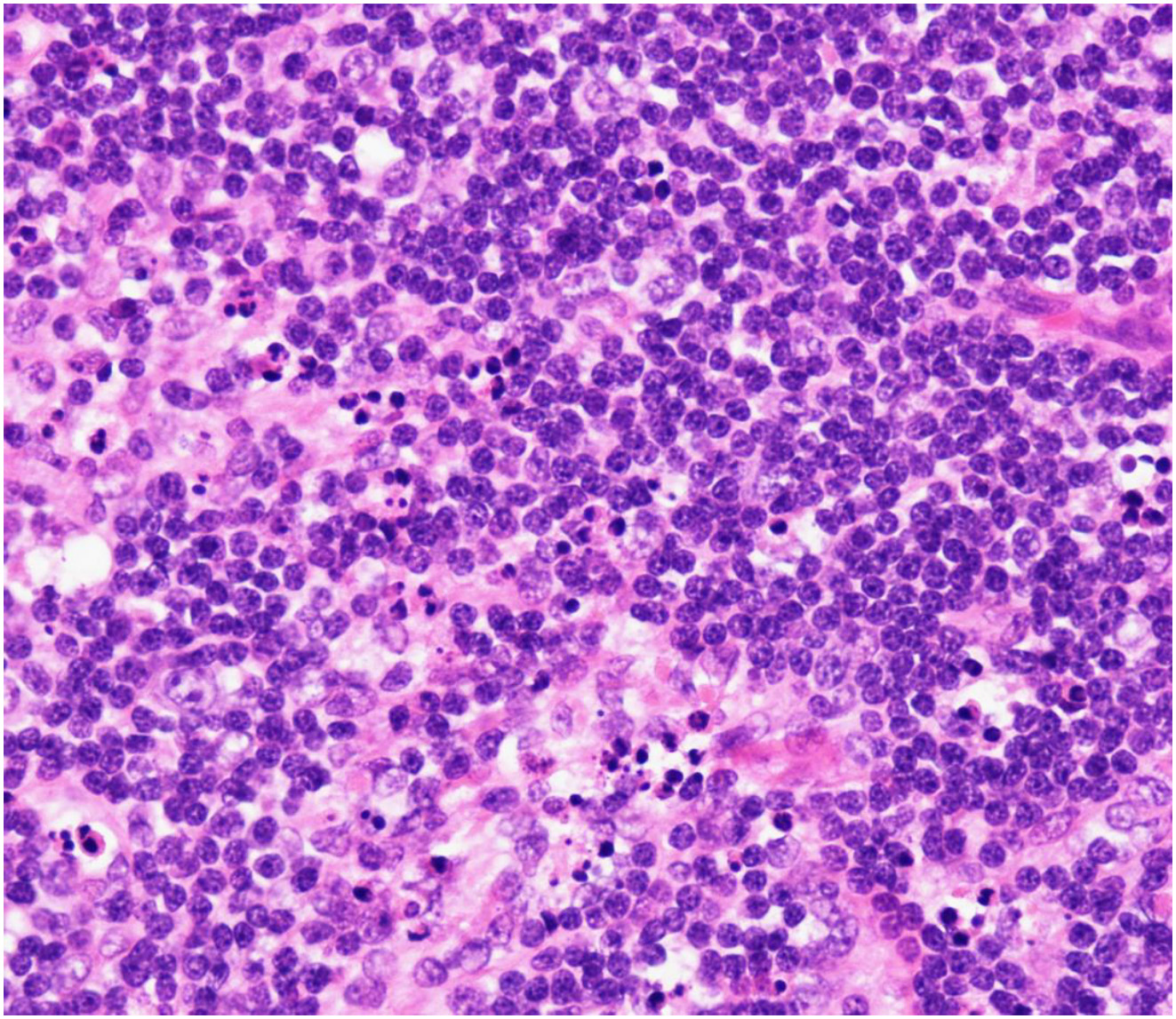
Pathological image of Kikuchi–Fujimoto disease

Lymph node involvement is usually cervical and unilateral in KFD, and bilateral cervical lymphadenopathy is rare.^1^, ^2^ Physicians should keep in mind that KFD can show bilateral lymphadenopathy like the present case.

## CONSENT

Written informed consent was obtained from the patient to publish this case report.

## AUTHOR CONTRIBUTIONS

AK and KO wrote the first draft and managed all of the submission process. HH supported writing of the first draft. FO organized writing of the manuscript.

## FUNDING INFORMATION

None.

## CONFLICT OF INTEREST

The authors declare no conflicts of interest.

## Data Availability

None.
